# L-glutaminase synthesis by *Klebsiella pneumoniae* (AS KP 23) isolated from clinical strain, and its efficacy against human hepatocellular and breast cancer cell lines

**DOI:** 10.1186/s12866-025-03773-3

**Published:** 2025-02-04

**Authors:** Lina J. Abdel-Hafez, Eman Y. T. Elariny, Asmaa E. Ibrahim, Mahmoud E. F. Abdel-Haliem

**Affiliations:** 1 Microbiology and Immunology Department, Faculty of Pharmacy, October 6 University, Cairo, Egypt; 2 Department of Botany and Microbiology, Faculty of Science, Zagazig University, Zagazig, 44519 Egypt

**Keywords:** L-glutaminase, Ammonium sulphate precipitation, Dialysis, Gel filtration chromatography, Breast cancer cell line, Hepatocellular carcinoma

## Abstract

**Background:**

Cancer is a major cause of morbidity and mortality worldwide. The proliferation of cancer cells depends largely on glutamine for survival and proliferation. Glutamine serves as a carbon source for the synthesis of metabolites and lipids via the TCA cycle, as well as a source of nitrogen for the synthesis of amino acids and nucleotides. Recently, the role of glutamine metabolism in cancer has been explored in many studies. Therefore, it provides a scientific relationship for targeting glutamine metabolism for cancer treatment. L-glutaminase which is a powerful anticancer medication that is widely used around the world, works by removing L-glutamine from cancerous cells. L-glutaminase has been cited as the most potent molecule that inhibits the proliferation of cancer cells, which significantly raises the possible applicability of cancer therapy and the possibility of its application as an alternative drug to chemotherapy. The first investigation into the antitumor property of L-glutaminase revealed its inhibitory effect on the growth of Gardner lymphosarcoma (6C3HED) and L-1210 leukemia cells. In the same study, glutaminase from *Pseudomonas spp.*, in combination with azaserine enhanced the degree of tumor growth inhibition. Subsequently, L-glutaminase was administered intravenously in patients with acute lymphoblastic leukemia and acute myeloid leukemia. Recently, a purified L-glutaminase from *Streptomyces sp*. D214 was shown to be the most effective, with an IC50 value of 10 mg/ml against the MCF-7 tumor cell line. Also, various in vitro studies have revealed that the activity of glutaminase against the proliferation of tumor cell lines using the MTT (3-(4,5- dimethylthiazol-2-yl)- 2, 5-diphenyltetrazolium bromide) cell proliferation assay. *Alcaligenes faecalis* KLU102 glutaminase was able to reduce the viability of HeLa cells in a dose-dependent manner, with an IC50 value of 12.5 mg/ml within 24 h.

**Results:**

In this study, a bacterium extracellular from human stool samples was extracted and identified using morphological, biochemical, and molecular methods. The 16 S rRNA gene was 100% identical to the sequence from *Klebsiella pneumoniae* and was submitted to GenBank under accession number OQ703039. Thus, this strain was named *Klebsiella pneumoniae* AS KP 23. Further kinetic studies on the purified enzyme were performed. In addition, the pH stability of the L-glutaminase enzyme was slightly affected over the pH range of 7.0–9.0 after 2 h of pre-incubation, and the rate of thermal inactivation of the L-glutaminase enzyme increased with higher temperatures and longer preheating periods. In addition, the stability of the tested enzyme decreased with an increasing storage period at -20 °C. The SDS-PAGE revealed that the L-glutaminase subunits had a molecular weight of around 97 kDa. L-glutaminase was purified 1.33-fold with a final specific activity of 799.9 U/mg protein using gel filtration chromatography. The enzyme´s cytotoxic activity showed severe toxicity against the HepG-2 human hepatocellular and breast cancer cell lines. *Klebsiella pneumoniae* glutaminase was able to reduce the viability of HeLa cells in a dose-dependent manner, with an IC50 value of 305.78 µg/ml in human hepatocellular carcinoma and an IC50 value of 400.51 µg/ml in breast cancer cell lines.

**Conclusion:**

*Klebsiella pneumoniae* AS KP 23 was a genetically determined microbial species isolated from human stool samples. The production of extracellular enzymes was examined. Additionally, purified L-glutaminase inhibited the growth of normal cells and showed potent anticancer activity against numerous cancer cell lines in the study. Its broad pH and temperature range, combined with its unique and highly stable catalytic activity, make it an excellent choice for use as an effective cancer inhibitor.

**Supplementary Information:**

The online version contains supplementary material available at 10.1186/s12866-025-03773-3.

## Background

Cancer of the liver (HCC) is the sixth most common kind of cancer worldwide [1]. In Egypt, it is the fourth most common cancer worldwide [2]. Numerous hospital investigations [3]. They have revealed a rise in the incidence of HCC. Hepatitis C virus (HCV) is the main risk factor for developing liver cancer, particularly HCC, and its prevalence and consequences are rising in Egypt [4]. Chemotherapy is one of several available cancer treatments. The limited efficacy and significant danger of the currently existing medications highlight the urgent need for new antitumor agents [5]. It is crucial to create novel therapeutic techniques with superior clinical research because a sizable fraction of patients endured significant clinical suffering for a prolonged duration, with risks of tumor recurrence and eventual metastasis [6]. Enzymes are versatile biocatalysts that play crucial roles in various biological and industrial processes, including pharmaceutical development, food technology, and environmental sustainability [7–10].

Recently, it has been shown that amino acid-depleting enzymes play a crucial role in the treatment of malignancies. L-glutamine is more likely to be used by these malignant cells than by the neighboring healthy cells for their development, proliferation, and energy requirements. The Achilles heel of a malignant cell is its inability to generate its amino acids since L-glutamine is also required as an essential nitrogen donor for the creation of pyrimidine and purine nucleotides in living cells [11].

The phosphate-activated enzymes known as L-glutaminases (EC 3.5.1.1) initiate the breaking of the side chain c-amide bond, which hydrolyzes glutamine into glutamic acid and ammonia. They belong to the broad family of amidohydrolases. It was discovered to only be connected to the inner mitochondrial membranes of hepatitis after being separated from all three domains of life, including prokaryotic and eukaryotic cells [12]. Since their synthesis is quick, inexpensive, mild, and compatible with the subsequent extraction and purification stages, microbial L-glutaminases have recently been preferred for various biotechnological applications, including pharmaceuticals and food makers. L-glutaminases come from a variety of sources, including plants, animals, and microbes. These microbes are preferred because of their ease of cultivation and large-scale economic production. Bacteria [13], fungi [14], and actinomycetes [15] make up the majority of microbial sources. Because of their diversity and unique characteristics, the amidohydrolase group of enzymes is most widely used as therapeutic enzymes. Due to their potential use in treating a variety of illnesses, such as acute lymphocytic leukemia and HIV, L-glutaminases have gained a lot of attention in recent years. Cancerous cells are better able to exploit L-glutamine for their energy needs, growth, and proliferation than the normal cells surrounding them by lowering the amount of L-glutamine in their environment. Since amidohydrolase L-glutaminase has been shown to have anticancer activity in the microbial system, many novel microbial sources have been attracted. Additionally, L-glutamine is a crucial nitrogen donor for the production of pyrimidine and purine nucleotides in live cells [16].

All living cells utilize the amide enzyme L-glutaminase, which is essential for cellular nitrogen metabolism [17]. The most recognizable enzyme that belongs to the family of -lactamases (serine-dependent) and penicillin-binding proteins is L-glutamine amidohydrolase (EC 3.5.1.2), which has a higher affinity for polymerizing and altering peptidoglycan biosynthesis, which is necessary for the synthesis of bacterial cell walls [18]. Additionally, the enzyme is classified as a proteolytic endopeptidase based on its catalytic efficiency since it cleaves peptide bonds and releases ammonia and glutamate as byproducts. According to Binod et al., L-glutaminase is essential for the absorption of nitrogen molecules and chemicals that are connected to them [19]. Additionally, L-glutaminase can be exploited in the food business as a flavor enhancer due to its unmistakable umami taste concentration [20]. Also, glutamine deprivation treatment inhibits tumor development preferentially by blocking the synthesis of new proteins and increasing the quantity of superoxide through oxidative stress, which encourages the death of cancer cells. L-glutaminase enzyme breaks glutamine down into glutamic acid and ammonia [21].

Plants, fungi, actinomycetes, and bacteria all have L-glutaminases. Several studies show that many microorganisms can produce laminases but at low concentrations. In the current study, we isolated, screened, and discovered *Klebsiella pneumoniae* AS KP 23, a bacterial extracellular L-glutaminase isolated from human stool. Species of *Pseudomonas*, *Vibrio*, *Bacillus*, *Moraxella*, *Aeromonas*, and *Acinetobacter* that produce L-glutaminase were also retrieved [22]. Concerning the occurrence of glutaminase producers in the environment, the majority of glutaminase-producing microorganisms were isolated from soil, except for a few in aquatic marine environments [23].

Different microbial resources with particular traits are still being researched in terms of effectiveness as a drug, productivity, and industry, along with optimizing biological processes using accessible materials and studying the kinetics of pure L-glutaminase [24]. L-glutaminase production has an economic problem that necessitates the search for new microbiological sources with high yields and the use of affordable, readily available resources for large-scale production [22]. Physical and nutritional conditions are crucial for the immersed fermentation of L-glutaminase production, depending on the presence of the appropriate substrate and end product-induced feedback inhibition [25]. This research focused on isolating l-glutaminase-producing *Klebsiella pneumoniae* AS KP 23 bacteria from the human stool of Zagazig University Hospital, Sharkia Governorate, Egypt, to enhance fermentation conditions, characterize the enzymes, and evaluate their efficacy against two cancer cell lines.

## Materials and methods

### Sample Collection, isolation, and identification of isolates

Out of 3 sources, canal water, sewage water, and human stool from Zagazig University Hospital, Sharqia, Egypt; 52 bacterial isolates were recovered during the period from Oct 2021 to March 2022. The samples were collected, they transferred immediately to the laboratories for culturing on blood agar, nutrient agar, and MacConkey agar, then incubated at 37ºC for 24 h. **Edwards and Ewing** [26] state that standard procedures were used for isolation, identification, and biochemical testing. The samples yield positive growth. Colonies are purified and used for biochemical identification tests. The rapid plate method was used to identify strains of the bacteria that produce L-glutaminase. The minimal agar media (KCl 0.5 g, MgSO_4_.7 H_2_O 0.5 g, Fe SO_4_.7H_2_O 0.1 g, Zn SO_4_.7H_2_O 1.0 g, KH_2_PO_4_ 1.0 g), L-Glutamine 0.5%, Phenol red 0.012 g, Aged Sea Water 1000 ml); The control plate was created without glutamine and used NaNO_3_ as the nitrogen source in its place. The prepared medium was autoclaved and then infected for 24 h. After that, the old bacterial colonies were cultured for 24 h at 37 °C. A pink zone encircled the bacterial colonies, and the zone index was calculated [27].

### Molecular characterization utilizing phylogenetic analysis and 16 S rRNA gene sequencing

DNA was extracted according to the QIA amp DNA Mini Kit instructions [28]. Using universal primers, the 16S rRNA gene was amplified and sequenced. Reverse primer [R1492] is 5’-GGTTACCTTGTTACGACTT-3’ while forward primer [F27] is 5’-AGAGTTTGATCCTGGCTCAG-3’ [29]. These primers enable the amplification of a segment of around 1500 bp by attaching to a region that is generally conserved. A Perkin Elmer Gene-Amp PCR system 9600 thermal cycler was used to carry out the PCR amplification. The following amplification circumstances applied: 35 cycles of denaturation at 95 °C for 30 s, annealing at 56 °C for 1 min, extension at 72 °C for 10 min, and 94 °C for 10 min. The method was used to detect the PCR products. The presence and quantity of PCR products (16 S rRNA gene) were assessed by electrophoresis on 1% agarose gel (Pharmacia Biotech, Denmark) [28]. The PCR product was purified using a GeneJETTM PCR Purification Kit (Fermentas). The amplified DNA fragment was partially sequenced using an ABI 3730xl DNA sequencer (GATC Biotech AG, Konstanz, Germany) with forward and reverse primers. The NCBI web server (www.ncbi.nlm.nih.gov) has received the 16 S rDNA sequences identified in this investigation. The Fundamental Local Alignment Search Instrument (BLAST) application (http://www.ncbi.nlm.nih.gov/blast) was utilized for analyzing the sequences and comparing them to published sequences [30]. The phylogenetic tree of the isolated bacteria was constructed based on 16 S rDNA data using Clustal W (http://www.clustal).

### Extraction and purification of L-glutaminase

Under ideal circumstances, the bulk-produced broth culture was refrigerated and centrifuged at 10,000 rpm for 10 min. to separate cell debris from the supernatant, also known as cell-free extract. The enzyme was precipitated by adding ammonium sulphate slowly (70%) and gently stirring continuously for the whole night at 4 °C. The mixtures were incubated overnight at 4 °C, and the precipitated protein was obtained by centrifugation for 20 min. at 6000 rpm. A minimum volume of 0.1 M citrate phosphate buffer with a pH of 8.0 was added to each fraction pellet for quick re-suspension. The precipitated mixture underwent dialysis with a Tris-HCl buffer (50 mM) overnight at 4 °C and submitted to a Sephadex G-200 column after centrifugation (4000 g for 20 min.). The major fraction was lyophilized after the L-glutaminase activity of the bound protein fractions was measured. Dissolved fractional precipitations were tested for enzyme activity and protein content [31].

### Gel filtration

Glutaminase was further purified on a Superose 12 h. column (1030 mm, Pharmacia Biotech) and adjusted to TE buffers (20mM Tris-HCl, 0.1 M NaCl, 10% ethylene glycol, 5mM EDTA; pH 7.5) at a flow rate of 0.3mL/min. for 1 h. Subsequently, the filtered fractions (5 mL) were loaded into the columns. After loading, the column was discharged into the TE buffer for 3 h at a flow rate of 0.2 mL/min. The collected fraction (1.5 mL) was examined for glutamate activity [32].

### SDS-PAGE analysis

The Biuret reaction, as reported by [33], was used to assess the protein content. According to the standard [34], the RJ sample was dissolved in 50 mL of sample buffer (62.5 mM tris-HCl (64.8 pH), 10 mM glycerol, 2 mM SDS, and 50 mM mercaptoethanol), pumped, and then heated at 95 °C for 3 min. 10 µl of protein were loaded onto a 10% SDS-PAGE acrylamide gel using a continuous current of 25 mA/gel for 70 min. after adding the blue bromophenol tracking dye (Universal Protein Electrophoresis System OmniPAGE Mini Vertical Protein, Cleaver Scientific Ltd, UK). 0.025 M Tris, 0.192 M Glycine, and 0.2% (w/v) SDS made up the running buffer. a 50 mA/gel continuous current for 3 h. After being washed with a high-speed shake of Coomassie brilliant Blue R-250 solution, the gel was then washed with a 4:1 mixture of methanol and acetic acid for dispersion to visualize the protein band. Gel Analyzer software (version 19.1) was used.

### Determination of enzyme activity

L-glutaminase activity was assessed as previously described by [35]. 0.5 mL of enzyme preparation, 0.5 ml L-glutamine (0.04 M), 0.5 mL phosphate buffer (0.1 M) (pH 8.0), and distilled water (0.5 mL) were incubated at 37 °C for 30 min. The reaction was stopped by adding 1.5 M trichloroacetic acid to 0.5 mL Once the combination had been kept at 20 °C for 20 min. at 450 nm in a spectrophotometer, 3.7 mL of distilled water, 0.1 mL of the aforementioned mixture, and 0.2 mL of a Nessler agent were added. The color was then read. As the controls, enzyme, and substrate blanks were utilized.

### Protein estimation

Using bovine serum albumin as the reference, the protein content in the crude enzyme source was calculated using Lowry’s technique [36] and the results were represented as mg/ml. The purified enzyme was characterized by its activity and stability at different temperatures (25–50 °C), pH (3.0–10.0), and reaction times (15–150 min). The effect of L-glutaminase concentration between 0. 1 M and 1.5 M on enzyme activity and L-glutaminase affinity to its substrate concentrations using different substrate concentrations of 0.1 M-2.0 M was also assayed. Additionally, at various temperatures (20–55 °C), the enzyme’s thermal stability was examined, and time intervals (30–150) min. for each temperature. The impact of the storage period was assayed by storing the purified L-glutaminase enzyme without its substrate at -20 °C for a period extended to 30 days. In addition, the amino acid composition of L-glutaminase was determined using HPLC.

### Cytotoxicity assay of L-glutaminase

This cytotoxicity activity test was carried out by the Regional Center for Mycology & Biotechnology at Al-Azhar University. The toxicity of the L-glutaminase generated by *Klebsiella pneumoniae* was assessed against (HepG-2 and MCF-7) human cancerous cells using the Sulphorhodamine B test (SRB). Different doses of the enzyme (15.6, 31.25, 62.5, 125, 250, and 500 µg/ml) were injected into each well of the microplate containing a known number (5 × 10^4^) after incubation of hepatocellular carcinoma cells (HepG-2) or Breast carcinoma cells (MCF-7). Fresh medium containing different concentrations of the test sample was added after 24 h of seeding. Serial two-fold dilutions of the tested chemical compound were added to confluent cell monolayers dispensed into 96-well, flat-bottomed microtiter plates (Falcon, NJ, USA) using a multichannel pipette. The microtiter plates were incubated at 37 ºC in a humidified incubator with 5% CO_2_ for 24 h. Three wells were used for each concentration of the test sample. Control cells were incubated without the test sample and with or without DMSO. After incubation of the cells at 37 °C, for 24 h., the viable cell yield was determined by a colorimetric method [37]. Then the cells were incubated for 24 h. The MTT test determined the number of viable cells. The optical density was measured at 590 nm with the microplate reader (SunRise, TECAN, Inc., USA) to determine the number of viable cells, and the percentage of viability was calculated as [(ODt/ODc)]x100% where ODt is the mean optical density of wells treated with the tested sample and ODc is the mean optical density of untreated cells. Cells from 80% of the convergence-growing cells were dissociated using trypsin and grown for 24 h. on a 96-well tissue culture plate before being treated with the enzyme. Compared to the control of untreated cells and, various enzyme treatments (0.01–1000 g/mL) were applied to the cells. The cells were exposed to the enzyme for 72 h. before being fixed with TCA (10% w/v) for an hour at 4°C. After multiple washing cycles, the cells were stained with a 0.4% (w/v) SRB solution for 10 min. in a dimly lit room. After the SRB-stained cells had dried overnight, they were dissolved in Tris-HCl, and the color intensity was measured at 540 nm in a microplate reader. The IC50 value was calculated using Sigma Plot 12.0 software by examining each tumor cell line’s percentage of viability in response to the different concentrations of the enzyme [38].

### Antimicrobial activity of the crude L-glutaminase enzyme

The agar well diffusion technique published by Turnidge and Paterson [39] was used to assess the isolated glutaminase enzyme’s antibacterial activity.

### Statistical analysis

The data were presented as mean + SD. Based on the information gathered from at least three separate trials (*n* = 3), mean values were determined. The student’s t-test was used for the statistical analysis. At P 0.05, the differences were deemed statistically significant.

## Results

### Isolation and screening of L-glutaminase-producing bacteria

In this study, fifty-two bacteria were isolated from different sources (canal water samples (5 isolates), sewage water (7 isolates), and human stool (40 isolates)). The rapid plate method was used to identify strains of the bacteria that produce L-glutaminase. A pink zone encircled the bacterial colonies that were proportional to their ability to produce L-glutaminase. From these isolates, only five isolates from human stool were able to form a pink zone in plates, which are characteristics of L-glutaminase-producing bacteria. The bacterial L-glutaminase hydrolyzed L-glutamine to glutamate and ammonia. The acid-base indicator dye phenol red converts to a pink color at a basic PH. The zone index was calculated for all L-glutaminase-producing samples. One of these strains, AS KP 23, demonstrated a detectable pink region surrounding the colony (22 mm), which was correlated with its capacity to manufacture L-glutaminase. A quantitative screening for L-glutaminase synthesis has been examined using submerged fermentation that tested spectrophotometry using Nessler’s method. The five bacterial species isolated were grown on synthetic liquid media containing 1% glutamine as the sole nitrogen source. 24-hour-old bacterial cultures were used as standard inoculum for each of the tested bacterial isolates. At the end of the incubation period, the bacterial cultures were filtered, and their filtrates were used for assays of L-glutaminase activity. The productivity of the L-glutaminase enzyme in bacterial filtrates was measured as the amount of ammonia (NH_4_^+^) released as a result of the enzyme reaction. The results presented in Table 1 showed that the highest activity of the L-glutaminase enzyme was obtained from the culture filtrate of *Klebsiella pneumoniae* (82.75 ± 4.71 U/ml crude enzyme). Following molecular identification, the high-potential L-glutaminase-producing strain AS KP 23 was selected for additional research.

**Table 1:** Screening of the selected isolated bacterial species for L-glutaminase production

Bacterial isolate	L- glutaminase activity(u/ml)
*Klebsiella pneumoniae*	82.75 ± 4.71^a^
*E.coli (1)*	56.2 ± 3.28^b^
*E.coli(2)*	42.6 ± 1.97^c^
*Acinetobacter junii*	21.5 ± 1.02^d^
*E.coli(3)*	15.7 ± 0.86^d^

### Biochemical and molecular characterization of AS KP 23

**Fig. 1:**
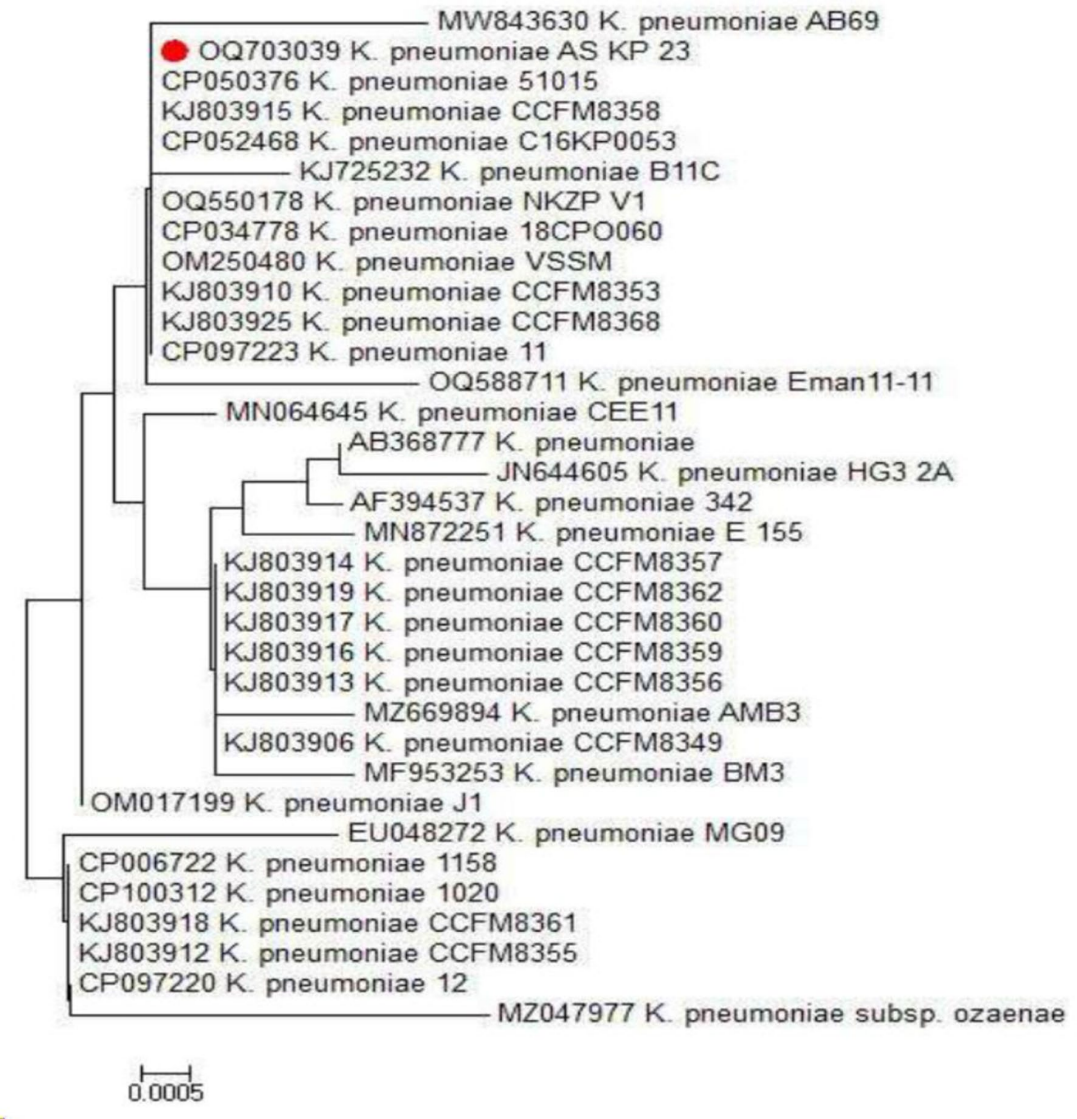
Phylogenetic tree of 16 S rDNA sequences similar to *Klebsiella pneumoniae*

AS KP 23 showed mucoid pink colonies on MacConkey agar. Then a gram-negative, short rod with a capsule was observed under a microscope. The Vogoes-Proskauer, Simmon’s citrate, and catalase tests were positive, but the methyl-red and indole tests produced negative results. On the Triple Sugar Iron (TSI) test, a yellowish slant was yellowish with no changes in the butt and no H2S produced, but a gas bubble appeared. Confirmed the identification by using phylogenetic analysis and 16 S rRNA gene sequencing using the universal primers F27 and R1492, the 16 S rRNA gene was successfully amplified by PCR to a length of ^~^1500 bp in supplementary Figure S1. A 699 bp piece of the isolated 16 S rRNA gene sequence was identified to support this identification. Upon comparison of the gene’s nucleotide sequences with similar sequences in the GenBank database using the Basic Local Alignment Search Tool (BLAST), it was discovered that isolate AS KP 23 had a 100% match with *Klebsiella pneumoniae* (Supplementary Figure S2). The sequence was submitted to the NCBI GenBank database with the accession number OQ703039, and the cluster analysis also showed its similarity to *Klebsiella pneumoniae* (Fig. 1).

### Purification of L-glutaminase enzyme produced by *Klebsiella pneumoniae* Precipitation of L-glutaminase enzyme by ammonium sulphate

The data presented in Table 2 show an overall increase in the purification fold of L-glutaminase by salting it out with ammonium sulphate compared with the crude preparations of the tested enzyme. The protein precipitated at this saturation showed a higher specific activity of *K. pneumoniae* L-glutaminase (618.96 u/mg) than the crude enzyme preparation (587.9 u/mg).

**Table 2:** Purification profile of *Klebsiella pneumoniae* L-glutaminase

Purification step	Volume (ml)	Total Protein (mg)	Total activity ux1000	Specific activity (u/mg)	Recovery (%)	Purification fold
Crude enzyme Ammonium sulphate Precipitation Dialysis	4000 200 50	2824 260.6 82	1660.3 161 63	587.9 618.96 779.9	100 9.7 3.8	1 1.1 1.33

### Dialysis

The dialysis process, as shown in the result, led to a rise in the specific activity of L-glutaminase up to 779.9 u/mg with an overall yield concerning the crude preparation.

### Gel filtration chromatography

**Fig. 2:**
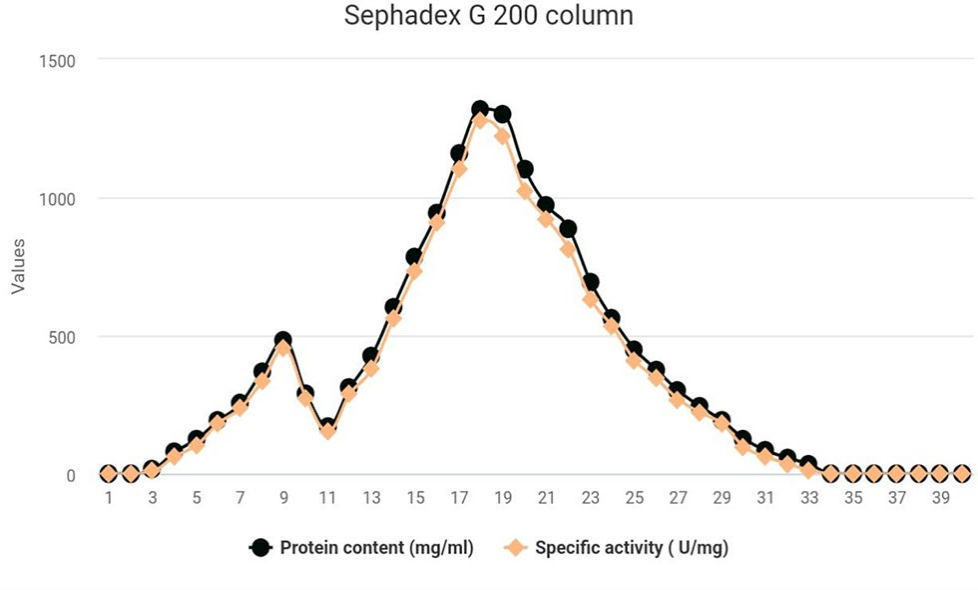
Gel filtration profile of crude L-glutaminase enzyme from the culture filtrate of *Klebsiella pneumoniae*

The protein content and L-glutaminase activity of each fraction were determined as described before for the tested enzyme. The results recorded and graphically presented in Fig. 2 show the complete profile of the gel filtration chromatography of the L-glutaminase system obtained from *Klebsiella pneumoniae*. The 40 protein fractions obtained from the column chromatography were separated into one molecular component of the extracellular L-glutaminase enzyme with two peaks.

### Gel filtration

**Fig. 3:**
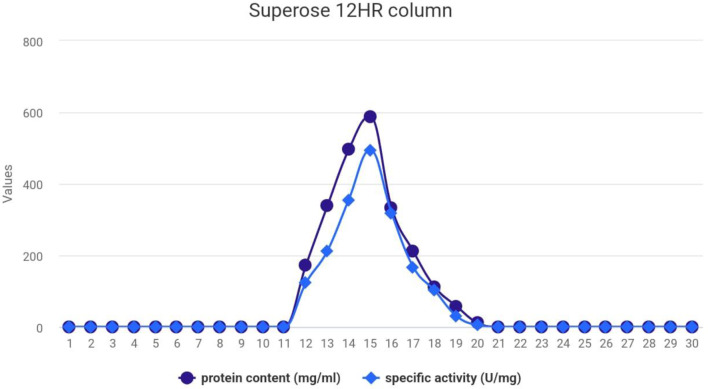
Gel filtration profile of crude L-glutaminase enzyme from the culture filtrate of *Klebsiella pneumoniae*

L**-**glutaminase was further purified in a Superose 12 h. column at a flow rate of 0.3mL/min for 1 h. The results recorded and graphically presented in Fig. 3 show the complete profile of the gel filtration chromatography of the L-glutaminase system obtained from *Klebsiella pneumoniae*. The 30 protein fractions obtained from the Superose 12 h. column were separated into one molecular component of extracellular L-glutaminase enzyme with one peak.

## Characterization of L-glutaminase enzyme

### SDS-polyacrylamide gel electrophoresis of the purified enzyme

**Fig. 4:**
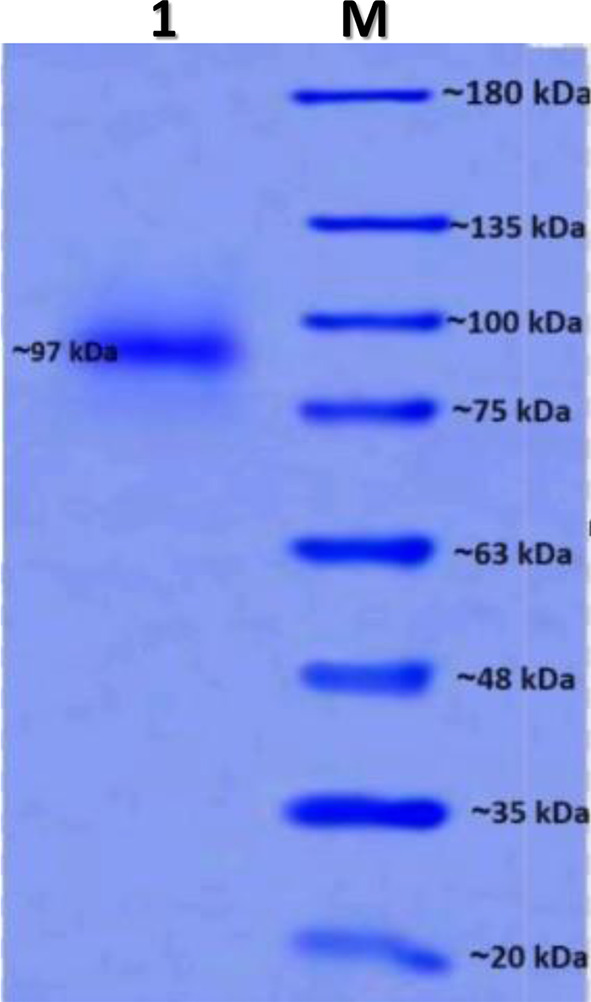
SDS-Polyacrylamide gel electrophoresis of the purified enzyme. M: protein markers; 1: Sephadex of purified enzyme

The result of SDS-PAGE analysis after the final step of purification revealed a single particular protein band corresponding to the molecular mass of approximately 97 kDa, which exhibited the purity of the enzyme (Fig. 4).

### Effect of enzyme concentration

**Fig. 5:**
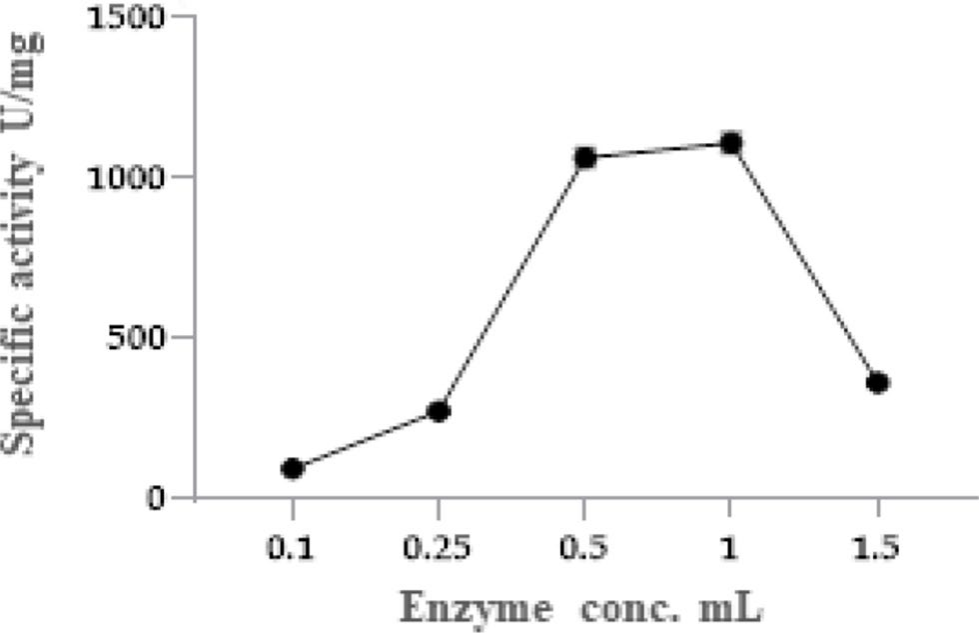
Effect of enzyme concentration on L-glutaminase activity from *Klebsiella pneumoniae*

The results, graphically illustrated in Fig. 5, revealed a parallel correlation between the concentration of the purified enzyme and the rate of substrate deamidation. It was observed that the specific activity of the L-glutaminase enzyme increased exponentially with the increase in enzyme concentration from 0.1 ml to the optimum value (1.0 ml) of *Klebsiella pneumoniae* glutaminase enzyme. The relationship became non-linear until a point was reached where either a plateau was observed or any further addition of the enzyme did not affect its activity. However, the specific activity of *Klebsiella pneumoniae* glutaminase shows its highest value (1108.8 u/mg protein**)** at 1.0 mL of the enzyme. Lower or higher protein concentrations lead to a decrease in the specific activity of the L-glutaminase enzyme.

### Effect of substrate concentration

**Fig. 6:**
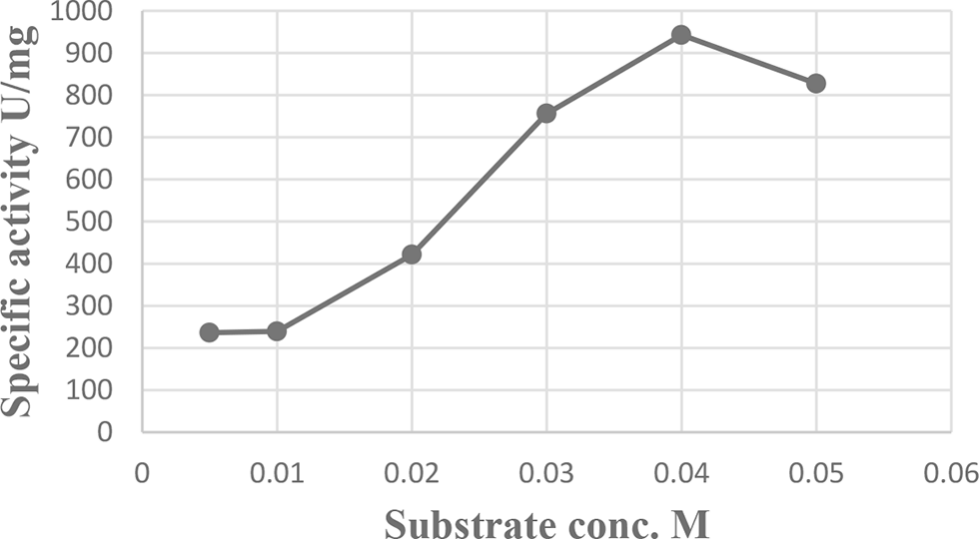
Effect of Substrate concentration on L-glutaminase activity from *Klebsiella pneumoniae*

The results are graphically illustrated in Fig. 6 showing that the optimum specific activity of L-glutaminase (942.9 u/mg protein) was determined at 1.5 mL substrate. Lower or higher substrate concentrations lead to a decrease in the specific activity of the L-glutaminase enzyme.

### Effect of reaction temperature (℃)

**Fig. 7:**
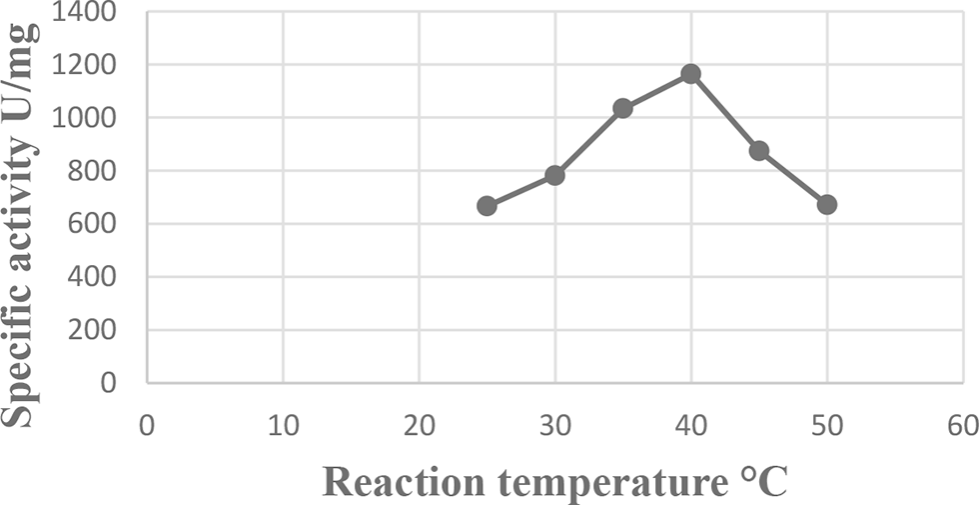
Effect of Reaction Temperature (℃) on L-glutaminase activity from *Klebsiella pneumoniae*

The findings, which are graphically shown in Fig. 7, show that the specific activity of the L-glutaminase enzyme rose concurrently with the rise in the reaction temperature, peaking at 40 °C (1163.9 u/mg protein), where it is at its highest level.

### Effect of reaction time

**Fig. 8:**
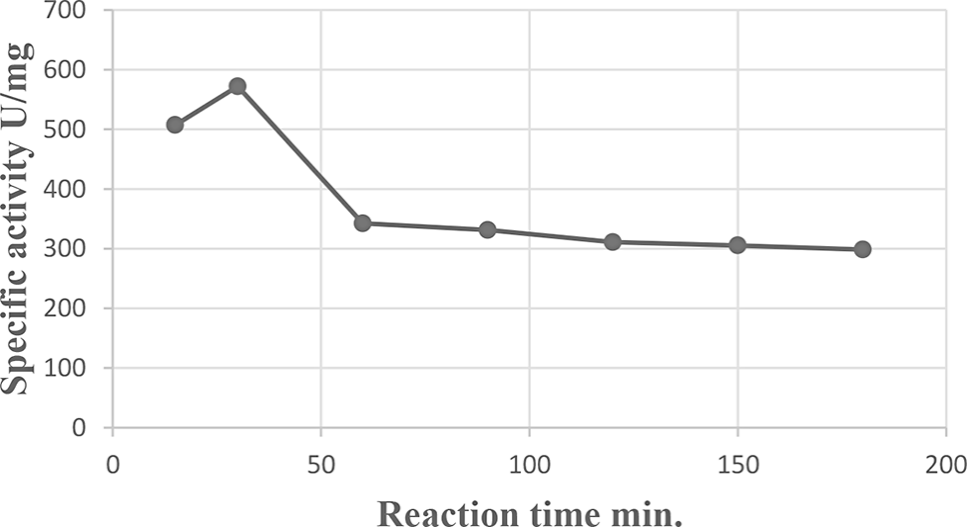
Effect of Reaction Time on L-glutaminase activity from *Klebsiella pneumoniae*

The results are graphically illustrated in Fig. 8 showing the reaction period that resulted in the highest level of enzyme activity. The greatest specific activity of the L-glutaminase enzyme (572.05 u/mg protein) was recorded after 30 min. On the other hand, further elongation of the reaction time after the optimum reaction time led to an inhibitory effect on L-glutaminase activity.

### Effect of pH values of the reaction mixture

**Fig. 9:**
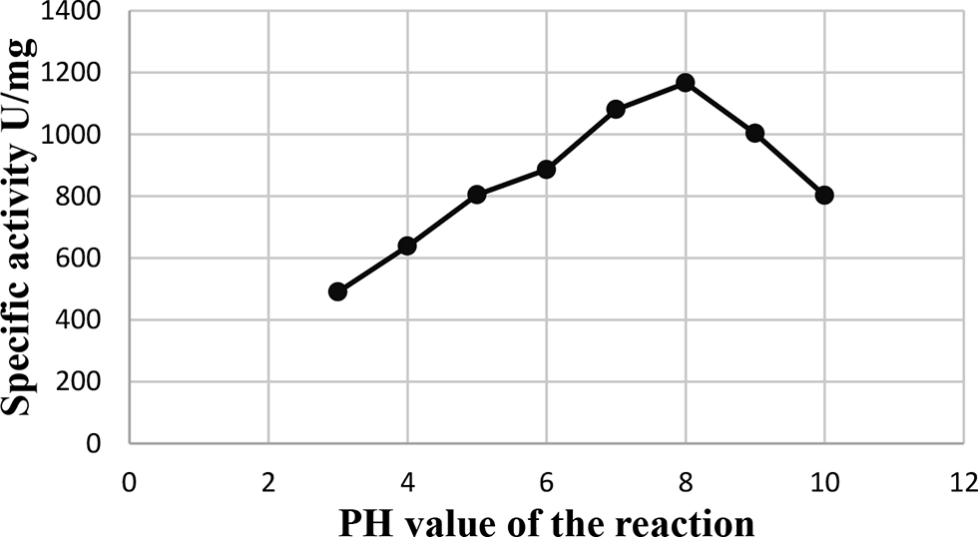
Effect of pH values of the reaction mixture on L-glutaminase activity from *Klebsiella pneumoniae*

The results shown in Fig. 9 show that the specific activity of L- glutaminase depends on the pH value. The specific activity increased gradually with increasing the pH value of the reaction mixture until it reached its optimum value at pH 8.0 (1166 u/mg protein).

### Effect of pH stability

**Fig. 10:**
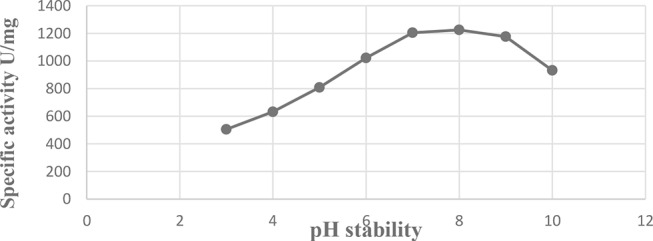
Effect of pH stability on L-glutaminase activity from *Klebsiella pneumoniae*

The findings are reported in Fig. 10, which demonstrate how closely L-glutaminase pH stability follows the pH that is ideal for L-glutaminase activity. After two hours of pre-incubation, the L-glutaminase enzyme’s pH stability throughout the pH range of 7.0 to 9.0 was marginally impacted.

### Effect of thermal stability

**Fig. 11:**
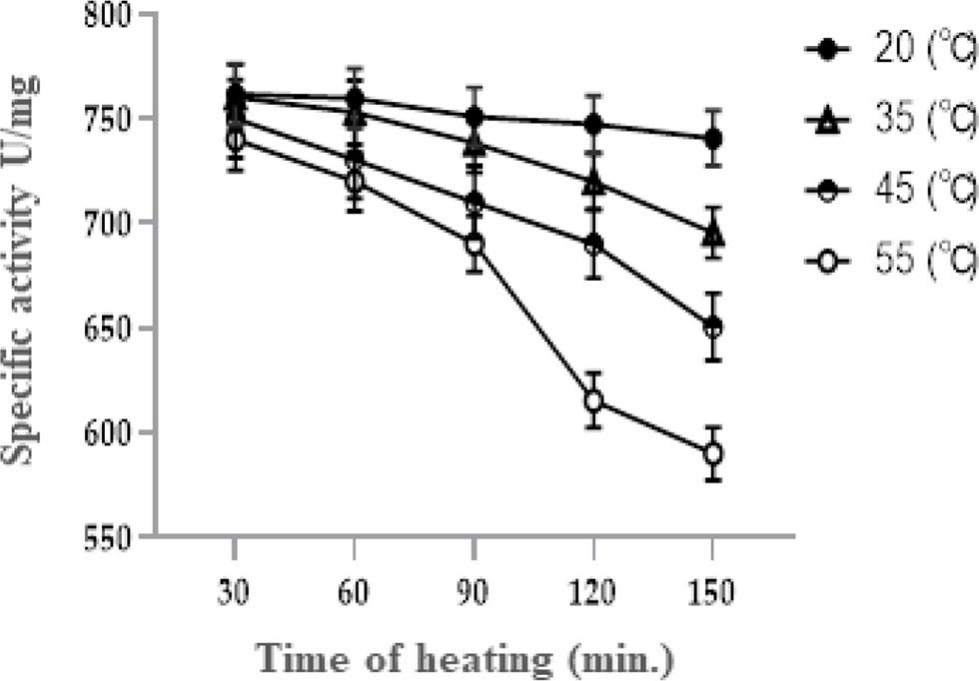
Effect of Thermal stability on L-glutaminase activity from *Klebsiella pneumoniae*

The findings, which are displayed in Table 3; Fig. 11, showed that the rate of thermal inactivation of the L-glutaminase enzyme increased with higher temperatures and long preheating periods. The preheating of the tested enzyme at 20 °C has no thermal inactivation effect on its activity. Consequently, at lower temperatures, the stabilizing effect of the tested enzyme was observed. On the other hand, L-glutaminase activity was significantly reduced by 77.4% by preheating at 55 °C for 150 min.

**Table 3:** Effect of thermal stability on L-glutaminase activity from *Klebsiella pneumoniae*

Temperature (℃)	Time of heating	Activity(u/ml)	Recovered activity (%)	Half-life (h)
20	30 60 90 120 150	761.8 759.59 750.96 747.37 740.62	100 99.7 98.6 98.1 97.2	48.6
35	30 60 90 120 150	760.33 752.93 738.48 719.96 695.52	99.8 98.8 96.9 94.5 91.3	45.6
45	30 60 90 120 150	750 730.4 710.2 690.1 650.5	98.5 95.9 93.2 90.6 85.4	42.7
55	30 60 90 120 150	740 720 690.3 615.4 590	97.1 94.6 90.6 80.8 77.4	38.7

### Effect of storage period at -20oC

**Fig. 12:**
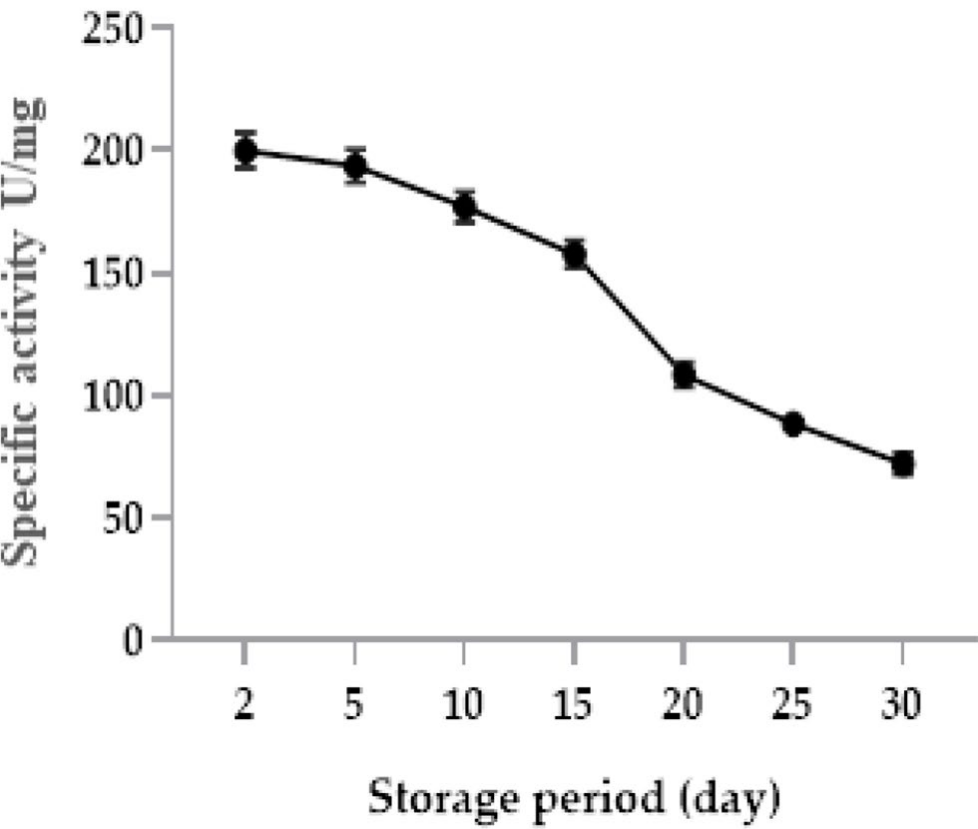
Effect of Storage period at -20^o^C on L-glutaminase activity from *Klebsiella pneumoniae*

The results presented in Table 4 and Fig. 12 show that the stability of the tested enzyme decreased with increasing storage period at − 20 °C.

**Table 4:** Effect of Storage period at -20^o^C on L-glutaminase activity from *Klebsiella pneumoniae*

Storage period (day)	Activity (u/ml)	Recover activity (%)	Half-life (day)
2 5 10 15 20 25 30	199.96 193.67 177 157.7 108.48 88.48 72.19	100 96.8 88.5 78.9 54.3 44.2 36.1	18.05

### Amino acids contents of the purified *Klebsiella pneumoniae* L-glutaminase enzyme

**Fig. 13:**
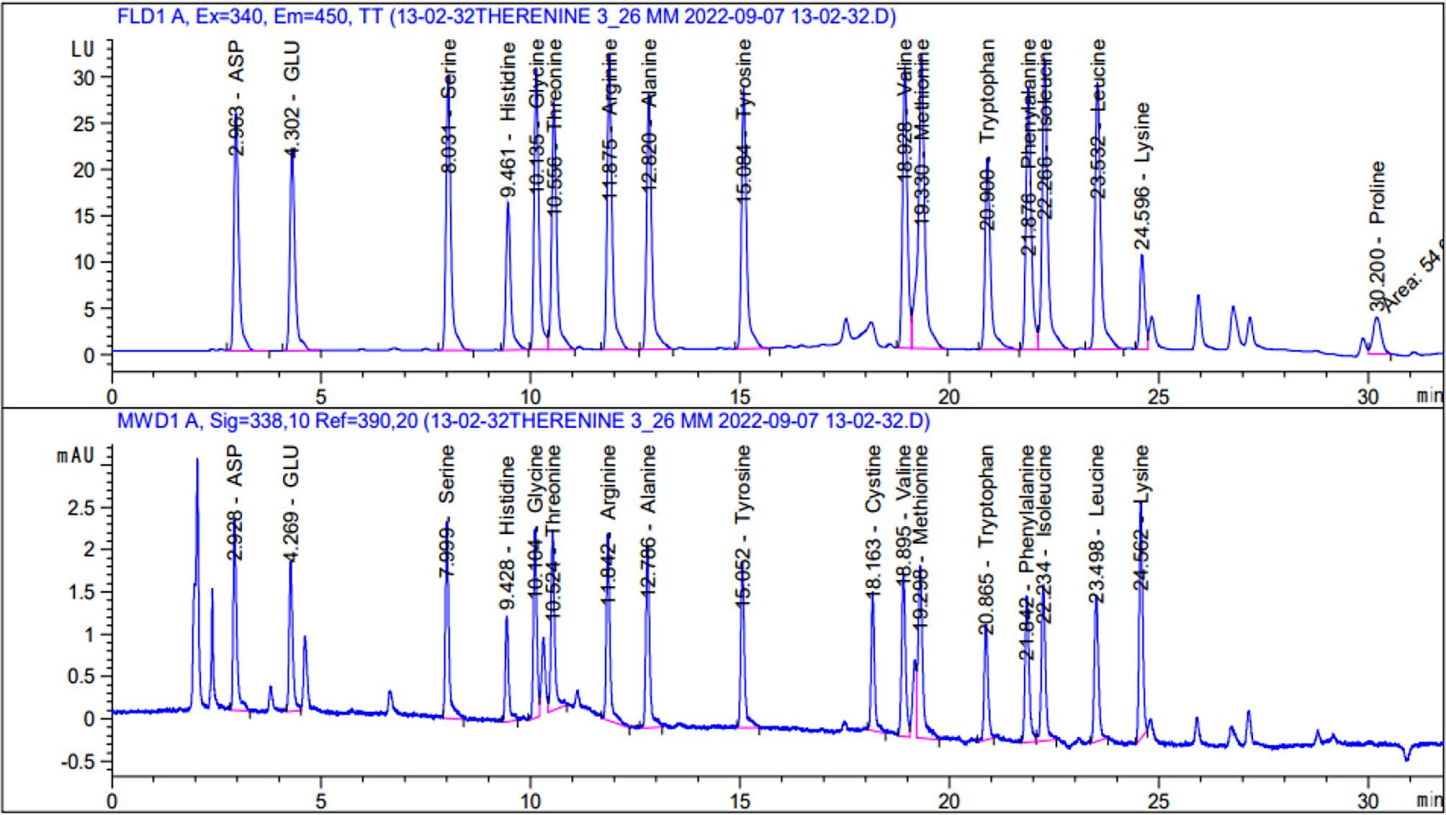
Amino acids contents of the purified *Klebsiella pneumoniae* L-glutaminase enzyme

The amino acid content of *Klebsiella pneumoniae*-purified L-glutaminase was studied. The results presented in Fig. 13 indicated that there are 17 amino acids in the *K. pneumoniae* L-glutaminase enzyme.

### Evaluation of cytotoxicity of L-glutaminase enzyme from *Klebsiella pneumoniae* against HepG-2 cell line

**Fig. 14:**
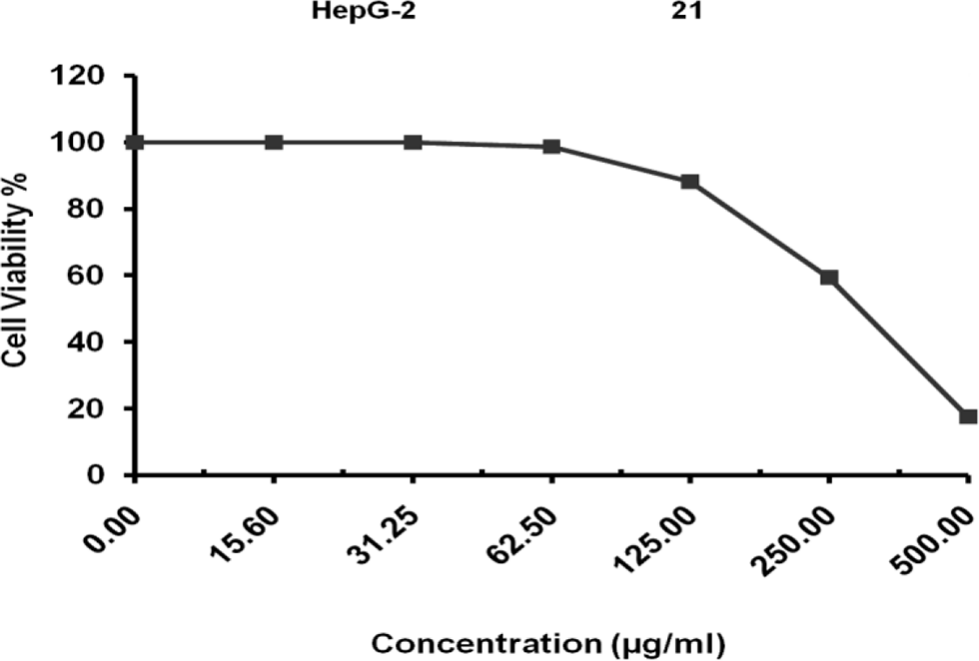
Cytotoxicity of L-glutaminase enzyme from *Klebsiella pneumoniae* against HepG-2cell line

The goal of the current investigation was to ascertain L-glutaminase’s in vitro cytotoxicity against the HepG-2 cell line. The Regional Center for Mycology and Biotechnology, Al-Azhar University, conducted the cytotoxicity activity test. The relation between surviving cells and drug concentration is plotted to get the survival curve of each tumor cell line after treatment with the specified compound. The 50% inhibitory concentration (IC50), the concentration required to cause toxic effects in 50% of intact cells, was estimated from graphic plots of the dose-response curve for each conc. The result in Fig. 14 shows that the half-maximal inhibitory concentration of cells IC50 was 305.78 ± 11.42 µg/ml.

### Evaluation of cytotoxicity of L-glutaminase enzyme from Klebsiella pneumoniae against MCF-7 cell line

**Fig. 15:**
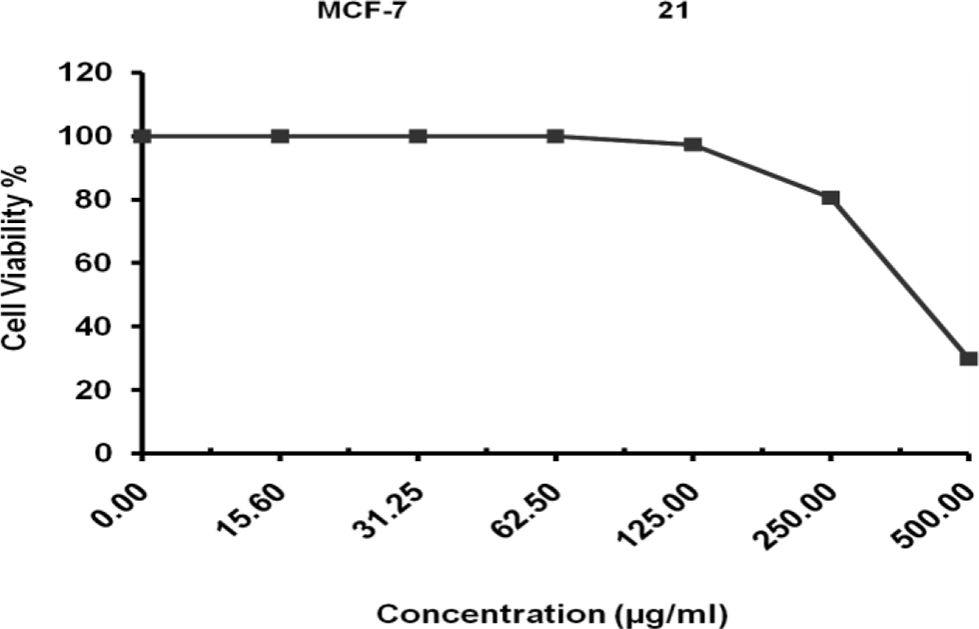
Cytotoxicity of L-glutaminase enzyme from *Klebsiella pneumoniae* against MCF-7 cell line

The current study’s objective was to determine L-glutaminase’s in vitro cytotoxic capability against the MCF-7 cell line. This cytotoxicity activity test was carried out by The Regional Center for Mycology & Biotechnology, Al-Azhar University. Results revealed that untreated control cells maintained 100% viability. However, treatment with L-glutaminase led to a dose-dependent reduction in cell viability, with an IC50 value of 400.51 ± 14.93 µg/mL as illustrated in Fig. 15. For comparison, the standard antileukemic drug Taxol (used as a positive control) had a significantly lower IC50 of 0.61 µg/mL.

### Antimicrobial activity of the crude L-glutaminase enzyme

The results in Table 5 show that the L-glutaminase enzyme from *Klebsiella pneumoniae* has poor antibacterial activity, as a crude enzyme preparation of concentration1000 µg/ml gives an inhibition zone with a diameter of 2 mm.

**Table 5:** Antimicrobial activity of the crude L-glutaminase enzyme

Conc. Of glutaminase(µg/ml)	Inhibition zone (mm)
125	0
250 500 1000	0 0 2

## Discussion

### Source of bacteria

It has been demonstrated that human stool is a significant source of microbial variety with the capacity to develop new bioactive substances such as beneficial bacteria present in the gut environment that can produce bacteriocins and prevent pathogenic microbes from colonization [40]. Various genera of probiotic bacteria can produce exopolysaccharides (EPSs) in large quantities [41]. Recently, microbial exopolysaccharides (EPSs) have a lot of attention due to their health benefits [42]. The exopolysaccharides from lactic acid bacteria have immunostimulatory activity, antitumor effects, antioxidant activity, and blood cholesterol-lowering ability [43]. Also, probiotic bacteria can carry out various metabolic activities due to their production of some enzymes such as lipases, esterases, and amylases [44]. Many bacteria produce B-group vitamins, that are soluble in water and absorbed into the intestine [45]. So, our findings demonstrated that bacterial isolates that generate L-glutaminase might potentially be found in the human feces environment, which encourages microbial diversity with exceptional, powerful enzymatic capacities [22]. Human feces samples included more L-glutaminase-producing bacteria than sewage and canal water samples.

### Screening and identification

The screening of the isolates was based on: the qualitative method described by Gulati et al. [27]. The change in the color of the medium from yellow to pink is an indication of extracellular L-glutaminase production by the colony. This color change can be due to a change in the pH of the medium, due to the breakdown of the amide bond in L-glutamine which liberates ammonia by the L-glutaminase enzyme. Phenol red at an acidic pH is yellow, and at an alkaline pH it turns pink; So, a pink zone is formed around the microbial colonies producing L-glutaminase. This results in agreement with *Bacillus* sp. DV2-37 [23]. Another method involves the isolation of microorganisms by routine isolation procedures from certain environments, which are then screened for enzymatic activity. However, the use of selective media and the presence of antibiotics, NaCl, and pH indicators make the MGA medium suitable for both direct and selective isolation of L-glutaminase-producing organisms [46].

AS KP 23 was grown into nutrient agar and MacConkey agar, respectively, by following Edwards and Ewing [26]. From cultural and gram staining tests, gram-negative, and rod-shaped, several different biochemical tests were performed to identify the isolate. The most common genomic indicator designed for confirmation of the identified bacteria molecularly is the 16 S rRNA gene, and it is thought to be a more precise way than more traditional ones. The 16 S rRNA gene was 100% identical to the sequence from *Klebsiella pneumoniae* and was submitted to GenBank under accession number OQ703039. Thus, this strain was named *Klebsiella pneumoniae* AS KP 23. The isolate that produced the greatest L-glutaminase, was AS KP 23. This bacterium species may have biotechnological uses that have previously been documented [27]. It may be possible to discover novel anticancer enzyme-producing microbial strains that have features that lessen the immunological response brought on by treatment. The 16 S rRNA gene is used to identify some bacterial strains that manufacture L-glutaminase such as *Bacillus cereus* MTCC 1305, *Aeromonas veronii*, *Acinetobacter calcoaceticus* PJB1 and *Halomonas* [47, 48]. The results show that the enzyme activity is 415.07 U/mL, the total protein content is 2824 mg, and the specific activity is 587.9 U/mg, which is considered to be the initial recovery of the enzyme activity after centrifugation of the extract and 100% assumed for subsequent studies.

The optimization of L-glutaminase production parameters, such as pH, temperature, incubation period, and nutrient sources, plays a crucial role in enhancing its potential as an anticancer agent. L-glutaminase has garnered significant interest due to its ability to deplete L-glutamine, an essential amino acid for the proliferation of cancer cells, particularly in glutamine-dependent tumors. The enzyme catalyzes the hydrolysis of L-glutamine to L-glutamate and ammonia, effectively starving cancer cells of a critical nutrient for their growth and survival. The synthesis of enzyme metabolic processes and the bioavailability of trace minerals are significantly influenced by the initial pH of the media [22]. In this study, we found that pH 8.0 is optimal for L-glutaminase production by *Klebsiella pneumoniae*, which aligns with the optimal pH range (7.0 to 8.5) reported for enzyme activity in other microorganisms [49]. The influence of pH on bacterial growth and enzyme production is crucial, as it directly affects the availability of metabolic ions and cell membrane permeability, both important factors in enzyme biosynthesis and stability. These findings are relevant to anticancer applications, as the stability and efficacy of L-glutaminase at physiological pH (close to 7.4) can enhance its therapeutic potential when administered in clinical settings. The optimal temperature for L-glutaminase production in this study was 40 °C, which is in agreement with other reports [48], and suggests that the enzyme retains significant activity at temperatures close to physiological conditions (37 °C). This is particularly important for anticancer applications, as maintaining enzyme activity within the human body’s temperature range ensures its effectiveness during therapeutic use. Due to the mesophilic character of the species, enzyme production is best at a temperature of 40 °C and decreases when microbial cultures are grown at temperatures above their maximum. The outcomes match those of *Bacillus subtilis* at 37 °C and pH 7.24 [50]. The observed enzyme production peak after 96 h of incubation indicates that extended fermentation can optimize yield before nutrient depletion and enzyme inactivation occur. Efficient production of L-glutaminase is critical for its use in large-scale biotechnological applications, including drug manufacturing for cancer therapy. After optimizing the fermentation process, the enzyme productivity of L-glutaminase of *Klebsiella pneumoniae* increased by 1.33 times. Optimum L-glutaminase production was recorded at pH 8.0, at a temperature of 40 °C, and after 96 h. of incubation. While L-glutaminase from *Brevundimonas*’ displayed its highest activity at 40 °C and pH 8.0 and after 28 h., maximal L-glutaminase synthesis started to decline [51], 48 h. of incubation of *Streptomyces griseus* [52] and 40 h. of *Bacillus cereus* MTCC 1305 [50]. As a result of the efficiency of bacterial growth and catalytic suppression of the finished product, glutamate, a lengthening of the fermentation time lowers enzyme production [53]. Nitrogen and carbon sources also significantly influenced L-glutaminase production. In our study, beef extract proved to be the best nitrogen source, which is consistent with other findings, and fructose was the most effective carbon source. The ability to enhance enzyme yield with specific nutrients supports the scalability of L-glutaminase production for pharmaceutical purposes [54, 55]. These optimized conditions for L-glutaminase production contribute to its potential application as an anticancer agent by ensuring high yield, stability, and activity in conditions relevant to therapeutic contexts. Further studies on its mechanism of action in cancer cells, along with clinical trials, are essential to fully harness the therapeutic potential of L-glutaminase in anticancer treatment.

In the case of *Bacillus sp*. DV2-37, glucose was found to be the best carbon source, L-glutamine was observed to enhance L-glutaminase synthesis (36.12 U/ml), the optimum L-glutaminase production was recorded at pH 7.0, and maximum L-glutaminase production was noticed at a temperature of 37 °C [23].

In the case of *Halomonas meridiana*, pH 8.0 was the most favorable for enzyme production (41.30 U/mL) and enzyme productivity, the optimum temperature for enzyme production was spotted up to 37 °C., glucose exhibited an enhanced effect for bacterial growth and enzyme production, and the yeast extract was the favored nitrogen source that enhanced L-glutaminase production [31].

In the case of *Halomonas hydrothermalis* B-15-9-2, the beef extract increases the enzyme activity from 3.45 ± 0.2 U/mL to 4.55 ± 0.4 U/mL and fructose is the most preferable carbon source which increases the enzyme activity [56].

The activity of L-glutaminase produced by *Fusarium solani-melongenae* was best at pH 8.0, with 0.980 U/mL, Starch was found to be a good carbon source compared to others resulting in maximum activity of 0.797 U/mL and maximum enzyme production on 7th day with activity of 0.665 U/mL [57].

### Enzyme purification

The purification protocol in this work includes successive analytical techniques to exclude undesirable proteins, as follows: precipitation of crude proteins by salting out with ammonium sulphate; dialysis via cellophane bag; gel filtration using column chromatography; a Sephadex G-200 column; and a Superose 12 h. column. Sodium dodecyl sulphate-polyacrylamide gel electrophoresis (SDS-PAGE) was performed, and the molecular weight of L-glutaminase was determined using standard molecular weight markers. In the same manner, L-glutaminase was partially purified by using the 70% ammonium sulphate saturation method, followed by dialysis, and purified by the column chromatography method using Sephadex G100 [58, 59]. Other investigators used ion-exchange chromatography for the purification of L-glutaminase, such as that produced by *Alcaligenes faecalis* KLU102 [60], and *Bacillus subtilis* OHEM11 [12], 75% ammonium sulphate was used [61], and 80% ammonium sulphate was used [62]. Also, gel filtration with a G-75 column was used [63]. Through the use of dialysis, Sephadex-200, and ammonium sulphate precipitation, the purity of the enzymes steadily improved [64].

SDS-PAGE has been used by many researchers to detect the purity of the L-glutaminase enzyme. Based on the source of the microorganisms (such as *Bacillus subtilis* OHEM11 (54.8 kDa) and *Bacillus cereus* LC13 (35.0 kDa), the optimal pH level and temperature of L-glutaminase activity are equivalent to the physical conditions of the human body, and *K. pneumoniae* L-glutaminase (kDa) has a variable molecular weight that is measured by SDS-PAGE examination [47]. On the other hand, the molecular weight of the glutaminase enzyme was 40 kDa [59], the molecular weight of L-glutaminase from *Streptomyces avermitilis* was 50 KDa [65], and the marine species *Halomonas meridian* produced L-glutaminase with a molecular weight of 57 kDa [31]. Most L-glutaminase enzymes have molecular weights ranging from 30 to 60 kDa [65].

### Characterization of enzyme activity

In the present study, the optimal substrate concentration for the activity of L-glutaminase was found to be 0.04 M and had the highest specific activity (942.9 u/mg protein). In the same way, the optimum substrate concentration for L-glutaminase activity was observed at 0.04 M [65, 66]. On the other hand, the optimum substrate concentration for maximum L-glutaminase activity was observed at 0.05 M [12].

In many investigations, the ideal temperature for maximal L-glutaminase activity was around 40–50 °C [59, 67, 68]. In our study, L-glutaminase also demonstrated thermal stability throughout a temperature range of 30 to 50 °C, maintaining 90% of its activity at that temperature. In the same way, L-glutaminase from *Bacillus sp.* DV2-37. showed thermal stability over a temperature range of 30–50 ^o^C and retained 90% of its activity at 60 °C [23]. In the case of *Bacillus subtilis* NRRL 1315, the enzyme still retained more than 58.44% of its optimal activity at elevated reaction temperatures from 45 to 60 °C, pointing out its good heat thermostability [66].

Additionally, according to the relationship between enzyme activity and pH, the glutaminases that have an optimum pH above neutral are appropriate for therapeutic use [69]. The purified *Klebsiella pneumoniae* AS KP 23 L-glutaminase activity peaked at pH 8.0 (1166 u/mg protein). Similarly, other studies of the optimal pH of the L-glutaminase enzyme were found to be in the alkaline range [31, 70, 71]. On the contrary, the optimum pH value of the purified L-glutaminase enzyme was 7.0 [23, 59].

### Cytotoxicity and Anticancer Potential

One of the most hazardous illnesses is cancer. It is the second-most prevalent illness among people [72]. There are various treatments, but the most efficient treatment is thought to be enzyme therapy. Enzymes are useful in cancer treatment because they are non-toxic, low-molecular-weight proteins with specialized actions. Enzymatic techniques have also been said to be more effective in treating cancer [73]. Enzymes can bind and work on their targets with remarkable affinity, then convert and catalyze a large number of target molecules into the desired products. Because of these two aspects, they are extremely particular and effective medications that can perform a specific therapeutic role in the body that other molecules cannot [74]. Furthermore, amidases deprive tumor cells of L-glutamine, resulting in the selective death of tumor cells that depend on L-glutamine [75].

The use of L-glutaminase-dependent deprivation treatment, which hydrolyzes L-glutamine into glutamate and ammonia and specifically suppresses tumor development by preventing the synthesis of new proteins, also raises levels of oxidative stress superoxides and encourages cancer cells’ demise [31]. As a result, it might be a candidate for enzyme treatment. In a variety of cancer types, L-glutaminase is being researched more and more as a potential antileukemia agent [12].

In this study, with an IC50 of 305.78 ± 11.42 µg/ml against HepG-2 and 400.51 ± 14.93 µg/ml against MCF-7, the isolated bacterium *Klebsiella pneumoniae* produced L-glutaminase with potential anticancer properties across all examined cell lines, showing promising activity compared to the untreated control. Since breast cancer is the second most frequent cancer globally, ongoing research focuses on identifying and screening potential therapeutic agents for its treatment [76].

### Comparative analysis with previous studies

The current study showed that the L-glutaminase-induced effects of *Klebsiella pneumoniae* are high in cell lines of hepatocarcinoma (HepG-2) and breast cancer (MCF-7) and associated with suppressing the progression of tumor cells. Our findings were in line with *Streptomyces rochei* SAH2_CWMSG L-glutaminase, which suppressed HepG2, MCF-7, and HCT-116 growth with IC50 of 279.7, 405.1, and 354.2 µg/ml, respectively [77], and *Streptomyces canarius* L-glutaminase anticancer activity, which was very active on the HepG2 (IC50, 6.8 g/mL) cell line, but ineffective against MCF7 cells [78]. L-glutaminase from *Bacillus cereus* MTCC gradually reduced the proliferation of hepatocellular carcinoma (Hep-G2) cell lines in the presence of various doses of L-glutaminase (10–100 µg/l) with an IC50 value of 82.27 µg/ml [69]. Furthermore, L-glutaminase of *Aspergillus flavus* displayed significant cytotoxicity against the two cell lines Hela and Hep G2, and the IC50 values for them were about 18 and 12 µg/ml, respectively, while the IC50 values for HCT-116 and MCF7 cells were 44 and 58 µg/ml, respectively [70]. The variations in cytotoxic effects among different L-glutaminases produced from different strains are related to the variation in glutamine levels in plasma throughout therapy. The level of glutamine reduction in cancer cells results in their death with varying efficacy, and this depends on the biological half-life of the animal, the kinetic properties of the enzymes, and the rate at which the amino acid enters circulation [78]. L-glutaminase from *Halomonas meridiana* showed the most potent cell apoptosis towards colorectal adenocarcinoma cells (LS 174T) with IC50 7 µg/mL. Concerning colorectal carcinoma cells (HCT 116), the enzyme showed a significantly promising cytotoxicity effect with IC50 13.2 µg/ml [31]. L-glutaminase produced by *Bacillus sp*. DV2-37 showed a potent cytotoxic activity of all cell lines in a dose-dependent manner. The results showed that MCF-7, HepG-2, and HCT-116 cell proliferation were significantly inhibited by L-glutaminase with IC50 values of 3.5, 3.4, and 3.8 µg/ml, respectively [23]. The toxic activity of *Pseudomonas sp.* RAS123 L-glutaminase against HCT-116 cells was significant with 83.51% inhibitory effect (IC50 value of 122 µg/ml), which was greater than the impact on HepG-2, MCF-7 (IC50 values of 175, 195, µg/ml, respectively), and a moderate inhibitory effect against HeLa (IC50 = 306 µg/ml), and a weak effect against CCL-86 (IC50 > 500 µg/ml) cells was noticed [79].

Few reports have been observed for the antimicrobial activity of L-glutaminase. L-glutaminase enzyme from *Klebsiella pneumoniae* has poor antibacterial activity, as a crude enzyme preparation of a concentration of 1000 µg/ml gives an inhibition zone with a diameter of 2 mm. On the contrary, the antibacterial activity of L-asparaginase and L-glutaminase-producing isolates with potent activity was examined, and the maximum antibacterial activity was observed against *Staphylococcus aureus*, *Pseudomonas aeroginosa*,* Shigella flexneri*,* Salmonella typhi*, and *Vibrio cholerae* [71]. In addition, the antimicrobial effect of *Pseudomonas sp*. RAS123 L-glutaminase showed activity against only bacterial strains (S. *aureus*,* B. subtilis*,* Streptococcus mutants*,* Enterobacter cloacae*,* and E. coli*) with inhibitory diameter zones ranging from 14 to 36 mm and no activity against fungal strains [79]. The antimicrobial activity of *Streptomyces griseorubens* was evaluated against different bacterial and fungal pathogens. Data showed that the enzyme has promising antimicrobial activity against *F. oxysporium*,* A. flavus*,* C. albicans*,* and S. aureus.* On the contrary, the enzyme showed no antimicrobial activity against *E. coli* [80]. The purified L-glutaminase from *Lactobacillus gasseri* BRLHM possessed significant antimicrobial activity against *Pseudomonas aeruginosa* isolates (*p* < 0.05), and the antibiofilm formation activity of the purified L-glutaminase was stronger than the antibiofilm activity of the referral standard drug, gentamicin (*P* < 0.05) [81].

To fully comprehend the therapeutic effectiveness of enzymes, more studies are required, including those on kinetic parameters, antigenic property reduction, half-life determination tests, and studies on enzyme stability, optimization of production conditions, pharmacologic profiles in animals, in vivo studies, potential clinical applications, and human clinical trials.

## Conclusion

The isolated microbial species from human stool samples were genetically identified as *Klebsiella pneumoniae* AS KP 23 and investigated the production of extracellular enzymes. This work has several intriguing elements, such as optimization variables, various cleaning techniques, molecular weight determination using SDS-PAGE, enzyme kinetic parameters, and substrate specificity. L-glutamine was purified using only three simple procedures, yielding a very pure band with a molecular weight of 97 kDa. Purified L-glutaminase also stopped normal cells from proliferating and had powerful antitumor action against many cancer cell lines in the research. It is ideally suited for usage as an efficient cancer inhibitor due to its very stable and distinctive enzyme catalytic activity, as well as its broad temperature and pH characteristics. However, there are some challenges to its use such as scaling up L-glutaminase production and maintaining enzyme stability, optimizing yield, and ensuring safety during clinical translation requires attention to unlock its full therapeutic potential. Furthermore, lymphocytes are dependent on glutamine metabolism which suggests that immunosuppression may be a side effect of drugs that target glutamine metabolism for cancer therapy, adverse effects like hypersensitivity reactions and its short half-life in vivo which reduces the duration of its action and require an intermittent supply by intravenous injection, which is expensive.

## Electronic supplementary material

Below is the link to the electronic supplementary material.

Supplementary Material 1

## Data Availability

The datasets utilized and/or examined in the present study can be obtained by contacting the corresponding author. Additionally, the genetic sequence of the strain analyzed has been submitted to the GenBank. The assigned accession number for the sequence is OQ703039, which can be accessed at https://www.ncbi.nlm.nih.gov/nuccore/OQ703039.
