# Supplementary material for: L-glutaminase synthesis by Klebsiella pneumoniae (AS KP 23) isolated from clinical strain, and its efficacy against human hepatocellular and breast cancer cell lines
Source: BMC Microbiol. 2025 Feb 4;25:62. doi: 10.1186/s12866-025-03773-3 (PMC11792699; doi:10.1186/s12866-025-03773-3)
Supplement: Supplementary file 1 — Supplementary Material 1 [file 12866_2025_3773_MOESM1_ESM.docx]

**Supplementary Fig. S1:** An 1% agarose gel electrophoresis showing PCR using DNA from isolates as a template amplified with forward primer and reverse primers. Lanes 1,2,3,4,5 and L (where 1= AS KP 23; 2, 3, 4, 5 = other bacteria). The expected size is ^~^1500 bp. L: GeneRuler^TM^ 1 Kb Plus DNA ladder.

Query 1 GCGTGGGGAGCAAACAGGATTAGATACCCTGGTAGTCCACGCCGTAAACGATGTCGATTT 60

||||||||||||||||||||||||||||||||||||||||||||||||||||||||||||

Sbjct 735 GCGTGGGGAGCAAACAGGATTAGATACCCTGGTAGTCCACGCCGTAAACGATGTCGATTT 794

Query 61 GGAGGTTGTGCCCTTGAGGCGTGGCTTCCGGAGCTAACGCGTTAAATCGACCGCCTGGGG 120

||||||||||||||||||||||||||||||||||||||||||||||||||||||||||||

Sbjct 795 GGAGGTTGTGCCCTTGAGGCGTGGCTTCCGGAGCTAACGCGTTAAATCGACCGCCTGGGG 854

Query 121 AGTACGGCCGCAAGGTTAAAACTCAAATGAATTGACGGGGGCCCGCACAAGCGGTGGAGC 180

||||||||||||||||||||||||||||||||||||||||||||||||||||||||||||

Sbjct 855 AGTACGGCCGCAAGGTTAAAACTCAAATGAATTGACGGGGGCCCGCACAAGCGGTGGAGC 914

Query 181 ATGTGGTTTAATTCGATGCAACGCGAAGAACCTTACCTGGTCTTGACATCCACAGAACTT 240

||||||||||||||||||||||||||||||||||||||||||||||||||||||||||||

Sbjct 915 ATGTGGTTTAATTCGATGCAACGCGAAGAACCTTACCTGGTCTTGACATCCACAGAACTT 974

Query 241 TCCAGAGATGGATTGGTGCCTTCGGGAACTGTGAGACAGGTGCTGCATGGCTGTCGTCAG 300

||||||||||||||||||||||||||||||||||||||||||||||||||||||||||||

Sbjct 975 TCCAGAGATGGATTGGTGCCTTCGGGAACTGTGAGACAGGTGCTGCATGGCTGTCGTCAG 1034

Query 301 CTCGTGTTGTGAAATGTTGGGTTAAGTCCCGCAACGAGCGCAACCCTTATCCTTTGTTGC 360

||||||||||||||||||||||||||||||||||||||||||||||||||||||||||||

Sbjct 1035 CTCGTGTTGTGAAATGTTGGGTTAAGTCCCGCAACGAGCGCAACCCTTATCCTTTGTTGC 1094

Query 361 CAGCGGTTCGGCCGGGAACTCAAAGGAGACTGCCAGTGATAAACTGGAGGAAGGTGGGGA 420

||||||||||||||||||||||||||||||||||||||||||||||||||||||||||||

Sbjct 1095 CAGCGGTTCGGCCGGGAACTCAAAGGAGACTGCCAGTGATAAACTGGAGGAAGGTGGGGA 1154

Query 421 TGACGTCAAGTCATCATGGCCCTTACGACCAGGGCTACACACGTGCTACAATGGCATATA 480

||||||||||||||||||||||||||||||||||||||||||||||||||||||||||||

Sbjct 1155 TGACGTCAAGTCATCATGGCCCTTACGACCAGGGCTACACACGTGCTACAATGGCATATA 1214

Query 481 CAAAGAGAAGCGACCTCGCGAGAGCAAGCGGACCTCATAAAGTATGTCGTAGTCCGGATT 540

||||||||||||||||||||||||||||||||||||||||||||||||||||||||||||

Sbjct 1215 CAAAGAGAAGCGACCTCGCGAGAGCAAGCGGACCTCATAAAGTATGTCGTAGTCCGGATT 1274

Query 541 GGAGTCTGCAACTCGACTCCATGAAGTCGGAATCGCTAGTAATCGTAGATCAGAATGCTA 600

||||||||||||||||||||||||||||||||||||||||||||||||||||||||||||

Sbjct 1275 GGAGTCTGCAACTCGACTCCATGAAGTCGGAATCGCTAGTAATCGTAGATCAGAATGCTA 1334

Query 601 CGGTGAATACGTTCCCGGGCCTTGTACACACCGCCCGTCACACCATGGGAGTGGGTTGCA 660

||||||||||||||||||||||||||||||||||||||||||||||||||||||||||||

Sbjct 1335 CGGTGAATACGTTCCCGGGCCTTGTACACACCGCCCGTCACACCATGGGAGTGGGTTGCA 1394

Query 661 AAAGAAGTAGGTAGCTTAACCTTCGGGAGGGCGCTACCA 699

|||||||||||||||||||||||||||||||||||||||

Sbjct 1395 AAAGAAGTAGGTAGCTTAACCTTCGGGAGGGCGCTACCA 1433

**Supplementary Fig. S2:** Pairwise comparison homology (100 %) between the sequences of the eluted PCR with 16S ribosomal RNA of *Klebseilla pneumoniae*
